# Evaluation of the Findings of Peripheral Blood Smear, Bone Marrow Aspiration and Biopsy, Iron Storage, and Immunophenotype in Patients with Chronic Lymphocytic Leukemia

**DOI:** 10.30699/IJP.2024.2011275.3170

**Published:** 2024-01-29

**Authors:** Shiva Didehban, Elham Jafari, Ali Hosseini, Parisa Khorasani Esmaili

**Affiliations:** 1 *Department of Pathology, Pathology and Stem Cell Research Center, Afzalipour Medical Faculty, Kerman University of Medical Sciences, Kerman, Iran*; 2 *Pathology and Stem Cell Research Center, Kerman University of Medical Sciences, Kerman, Iran*

**Keywords:** Bone marrow cells, Chronic lymphocytic leukemia, Lymphocyte Immunophenotyping

## Abstract

**Background & Objective:**

Chronic lymphocytic leukemia (CLL) is one of the most common types of leukemia in adults with various signs, symptoms, and types of progression. In this study, we have investigated the frequency and correlation of laboratory findings including peripheral blood smear, bone marrow aspiration and biopsy, and cellular immunophenotyping in CLL patients.

**Methods:**

In this cross-sectional and retrospective study, the laboratory information of all 161 patients with definite diagnoses of CLL was extracted, and the frequency and correlation between different laboratory data were analyzed by descriptive statistics methods and Jamovi software version 2022.

**Results:**

Demographic factors such as age and gender, and laboratory factors such as anemia, thrombocytopenia, white blood cell count, percentage of lymphocytes, and patterns of bone marrow involvement were evaluated for 161 patients. There was a significant relationship between the bone marrow iron storage and the percentage of FMC7 marker expression with the percentage of atypical lymphocytes in the peripheral blood.

**Conclusion:**

Chronic lymphocytic leukemia, a prevalent form of leukemia associated with substantial mortality and morbidity, can be detected through a range of diagnostic techniques. Analyzing the results of these diagnostic tests and examining the prevalence of these indicators in patients afflicted with the condition can prove highly beneficial for prompt disease diagnosis, and prognosis determination among affected individuals.

## Introduction

Chronic lymphocytic leukemia (CLL) is one of the most prevalent types of leukemia, especially in Western countries. This disease is a clonal lymphoid malignancy characterized by the accumulation of mature B lymphocytes in the blood, lymph nodes, spleen, liver, and bone marrow (1-3). The disease mainly affects the elderly and male gender (4). According to studies, chronic lymphocytic leukemia accounts for more than 30% of leukemias in the world (5). Up till now, there have been no precise records regarding the occurrence of CLL in Iran. However, based on available evidence, it can be inferred that chronic lymphocytic leukemia, along with acute lymphocytic leukemia, acute myeloid leukemia, and multiple myeloma, are frequently encountered types of blood malignancies in Iran. Leukemias collectively contribute to approximately 8% of all malignancies identified in the country (6-8). 

The variety of clinical manifestations observed in individuals diagnosed with chronic lymphocytic leukemia varies greatly. Nowadays, the growing demand for complete blood cell count (CBC) tests has resulted in the early detection of this condition. As a result, more than 70% of patients are diagnosed in the early stages of this disease (the early stages of the Rai and Binet classification system, which is explained below). However, the progression of the disease in these individuals varies, and about 30-40% of them experience rapid advancement of the disease and clinical symptoms (9, 10).

Various methods are employed to evaluate the prognosis of chronic lymphocytic leukemia, one of the most renowned among them being the Rai and Binet classification system. This system relies on clinical parameters like CBC and physical examinations, as well as the number of involved organs. The Rai system comprises five levels (0 to IV), while the Binet system consists of three levels: A, B, and C. Additionally, there is a modified Rai system that features three risk levels—low, intermediate, and high. These correspond to levels 0 and combinations of Level I and II, as well as a combination of Level III and IV from the Rai classification system (Table 1) (11, 12).

**Table 1:** Rai and Binet system for CLL classification

Rai stage		Binet stage	
0-	Lymphocytosis only	A	<3 Lymphadenopathies plus Lymphocytosis
I-	Stage 0 plus Lymphadenopathy	B	≥3 Lymphadenopathies plus Lymphocytosis
II-	Stage 0 plus Splenomegaly or hepatomegaly		
III-	Stage 0 plus Anemia (hemoglobin <11 g/dL)	C	As for stage A or B Hemoglobin<10g/dL and/or platelet count <100 × 10^9^/L
IV-	Stage 0 plus Thrombocytopenia (platelet count <100 × 109/L)		

Various laboratory assessments have been proposed for diagnosing chronic lymphocytic leukemia. These methods encompass the complete blood cell count test (CBC), peripheral blood smear (PBS), flow cytometry (cell immunophenotyping), bone marrow aspiration and biopsy (BMA/B), cytogenetic tests, fluorescent in situ hybridization (FISH) test, molecular tests, lymph node biopsy, spinal tap, and other diagnostic procedures. Among these, peripheral blood smear and cellular immunophenotyping hold greater significance (13).

**Fig. 1:**
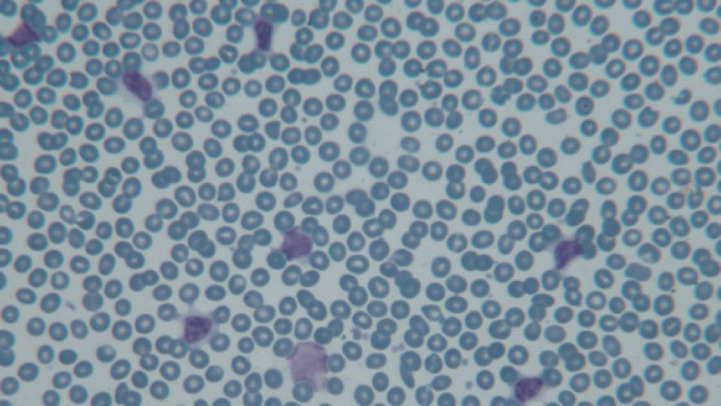
Presence of atypical lymphocytes, prolymphocytes, and smudge cells in peripheral blood smear (Giemsa, 400X magnification)

Peripheral blood smear: The first and most important change that occurs in patients with CLL is lymphocytosis in the peripheral blood and bone marrow. Classic 'smudge cells' or 'basket cells' are also observed in the peripheral blood smear (14) (Figure 1).

In the 5th edition of the WHO classification of hematolymphoid tumors, the family of pre-neoplastic and neoplastic small lymphocytic proliferations under the section of mature B-cell neoplasms consists of two entities: Monoclonal B-cell Lymphocytosis (MBL) and Chronic Lymphocytic Leukemia/Small lymphocytic Lymphoma (CLL/SLL) which remains the same and the category of 'B-prolymphocytic leukemia' (B-PLL) is no longer used, as it includes a heterogeneous group of hematolymphoid neoplasms (15).

The MBL group contains a subtype of CLL/SLL-type MBL with monoclonal CLL/SLL-phenotype B-cell count ≥0.5 x 10^9^/L and total B-cell count less than 5 x 10^9^/L with no other features of CLL/SLL (15).

Patients with CLL can be classified into several categories based on cell morphology in peripheral blood smears. The first category is typical CLL, in which the percentage of prolymphocytes and atypical lymphocytes is at most 10%. Another group with a disease similar to CLL consists of patients whose peripheral blood prolymphocyte percentage ranges from 11 to 55%; this condition is termed CLL/PLL (prolymphocytic leukemia). If the percentage of prolymphocytes exceeds 55%, the person is diagnosed with PLL (16).

The next category encompasses patients whose percentage of peripheral blood atypical lymphocytes (lymphoplasmacytic and cleaved cells) exceeds 10%; they are grouped as Atypical CLL which is not included in the 5th edition of the WHO classification of hematolymphoid tumors (17). Evidence supports a strong relationship between clinical symptoms, disease prognosis, and the type of CLL. Patients with atypical cell morphology CLL (both CLL/PLL and Atypical CLL) experience a worse prognosis and disease progression compared to those with typical CLL (16, 18).

Complete blood count test (CBC): Typically, a common finding in this test is a high white blood cell (WBC) count with a predominance of lymphocytes. Anemia and thrombocytopenia are also other important findings from this test. If present, they indicate the progression of the disease (19).

Bone marrow aspiration and biopsy: Their findings can assist in accurately diagnosing the disease and determining the appropriate treatment. In CLL patients, it is typically observed that lymphocytes make up more than 30% of bone marrow cells, exhibiting the same morphology as blood lymphocytes. Moreover, these cells generate diverse morphological patterns in the bone marrow, including diffuse lesions, interstitial patterns, and aggregations in the form of nodules (20).

**Flow Cytometry and Immunophenotyping**

These diagnostic tests are employed to confirm the diagnosis of CLL, assess the specific cell types involved, and determine the disease prognosis. The typical CLL phenotype often includes being CD5(+), CD19(+), CD20(+), CD23(+), FMC7(-), with weak expression of surface Ig (sIg), and weak expression or absence of membrane CD22 (4, 14, 21-23).

Bone marrow iron storage: This is one of the quantities assessed in the bone marrow and graded on a scale from 0 to 5 using Prussian blue staining (16). Currently, there are no conclusive studies on the level of bone marrow iron storage and its diagnostic value in patients with hematologic malignancies, such as CLL.

Assessing the results of these diagnostic tests and examining the occurrence of these findings among patients can greatly aid in the prompt diagnosis of the disease. This, in turn, enhances the treatment procedure and diminishes the mortality and morbidity rates among these patients. Consequently, in this study, we have undertaken the task of examining the frequency and significance of findings from peripheral blood smears, bone marrow aspirations and biopsies, iron storage evaluations, and immunophenotyping in individuals diagnosed with chronic lymphocytic leukemia.

## Material and Methods

This study was conducted in a cross-sectional and retrospective epidemiological manner, including all patients (161 patients) diagnosed with chronic lymphocytic leukemia, who were referred to the hematopathology department of Afzalipur Hospital in Kerman, Iran from 2015 to 2022. Diagnosis was established through clinical history, physical examination, complete blood cell count, peripheral blood smear, bone marrow biopsy and aspiration, immunophenotypic criteria, and a monoclonal B lymphocyte count ≥5×10^9^/L as per the IWCLL (International Workshop on Chronic Lymphocytic Leukemia) guidelines the same as the 5th edition of the WHO classification of hematolymphoid tumors (15).

While atypical CLL is not included in the 5th edition of the WHO classification, our study categorized patients into two groups—typical and atypical chronic lymphocytic leukemia and compared various factors between these two groups—due to its retrospective nature. This decision was also prompted by Robak *et al.*, who discussed atypical CLL in their study despite explaining the exclusion of the atypical category in the last version of the WHO classification (17). The classification was determined by the presence of atypical lymphocytes exceeding 10%, prolymphocytes exceeding 10%, or the expression of markers FMC7 exceeding 20%, CD23 below 20%, CD5/19 below 20%, and instances of CD20 bright expression, which were considered indicative of Atypical CLL cases. A comparative analysis was conducted between the two groups, examining bone marrow involvement patterns, bone marrow iron storage, and marker expression.

**Inclusion Criteria**

Confirmation of CLL diagnosis through clinical history, physical examination, complete blood count, peripheral blood slide, bone marrow biopsy and aspiration, immunophenotypic criteria, and a B lymphocyte count ≥ 5x10^9^/L.

Inclusion of newly diagnosed CLL cases that have not received prior CLL treatment.

Availability of patient's diagnostic test information, including peripheral blood smear report, complete blood cell count, bone marrow aspiration and biopsy report, and immunophenotyping report of the disease, in the information archive of Afzalipur Hospital, Kerman.

**Exclusion criteria:**

Patients without a definitive diagnosis of CLL will be excluded from the study.

Patients with other B and T lymphoid neoplasms were also excluded from the study.

The study was retrospective, and information related to it was extracted from the flow cytometry and pathology sheets of patients available in the hematopathology laboratory of Afzalipur Hospital. However, due to the incomplete availability of information in the main patients’ files in the hospital archive regarding patients' history and physical examinations, and considering that some patients were diagnosed with Chronic Lymphocytic Leukemia (CLL) on an outpatient basis, our access to certain details was limited. Consequently, information such as the presence of lymphadenopathy, and organomegaly was not included in this study. Furthermore, since the patients included in this study were newly diagnosed cases of CLL and due to the retrospective cross-sectional nature of the study, no follow-up evaluations for Richter's transformation were conducted.

Data collection was carried out using a registration form that encompassed various components. This form included demographic information of patients, comprising age and gender. Furthermore, it gathered information from peripheral blood smear examinations, covering the assessment of red blood cells, their morphology, and white blood cells along with their differential and count, as well as platelets. Additionally, it encompassed data acquired from biopsies and bone marrow aspirations, involving the categorization of factors such as cellularity, bone marrow involvement pattern, bone marrow iron storage, and bone marrow cellularity. Lastly, the immunophenotypic details of lymphocytes were also documented. All these aspects were meticulously extracted for each patient from pathology and flow cytometry reports. The accumulated data were then encoded into an Excel file for subsequent analysis and review.

For statistical analysis, Jamovi version 2022 software was utilized, with P values of less than 0.05 being considered significant.

The code of ethics for this study was IR.KMU.AH.REC.1401.190.

## Results

**Fig. 2:**
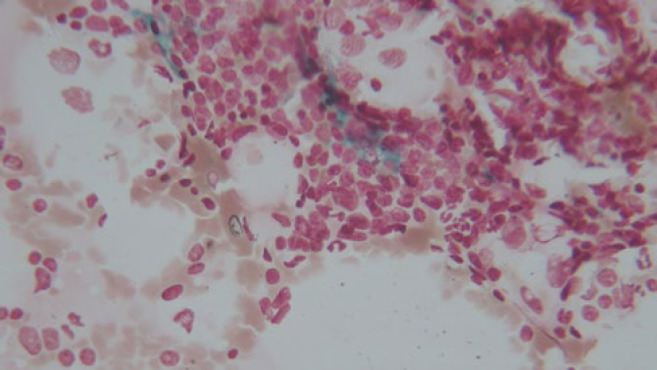
Bone marrow iron storage (grade 3) (Prussian blue, 400X magnification)

**Fig. 3:**
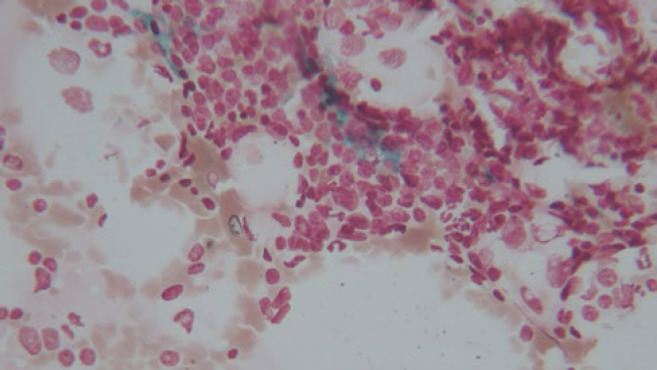
A. Increased number of small to medium-sized lymphoid cells with scattered prolymphocytes in bone marrow aspiration (Giemsa, 400X magnification), **B.** Nodular pattern of involvement in bone marrow biopsy (Hematoxylin & Eosin, 100X magnification)

In the descriptive findings section, the results are as follows:

Among the 161 patients with CLL, 93 individuals were in the Atypical CLL category, and 68 individuals were in the Typical CLL category. Out of the 161 patients with CLL, 50 were female, and 111 were male. Among the 158 patients with CLL at the time of diagnosis, 94 had anemia, while 64 did not have anemia. Of the 160 patients with CLL, 31 had thrombocytopenia at the time of diagnosis, and 129 had normal platelets. The number of patients with CLL was 7, 27, 37, 35, 19, and 6, categorized according to the degree of iron storage from grade 0 to 5 (Figure 2). The frequency of patients for each pattern of bone marrow involvement was as follows: 26 had the interstitial pattern, 8 had the focal pattern, 75 had the diffuse pattern, 21 had the nodular pattern, 12 had the interstitial & nodular pattern, and 17 had the interstitial & focal pattern (Figure 3) (Up to 161 patients, the remaining number of patients had missing data). Other descriptive results are listed in Table 2.

**Table 2:** Distribution of descriptive results of various analyzed factors

	Mean	Median	Standard deviation	Minimum	Maximum
Age	63.8	64	10.8	30	89
WBC count	50928	30000	61371	6300	500000
Lymphocyte percent	55.9	62	28.2	0	100
Lymphocyte count	27628	14040	39716	0	277200
Atypical lymphocyte (%)	20.4	11	24.5	0	85
Prolymphocyte (%)	3.33	0	5.23	0	25
Platelet count	140101	130000	70703	10200	340000
FCM/CD19 expression (%)	57	57	20.5	7	95
FCM/CD19&CD5 co-expression	43.6	41.1	18.4	14	94
FCM/CD22 expression (%)	32.6	30.7	23	0.1	91.8
FCM/CD20 expression (%)	52.1	49.3	20.8	9	100
FCM/CD23 expression (%)	47.7	52	20.7	0.36	93
FCM/FMC7 expression (%)	6.85	4	12.2	0.02	100

FCM: Flow cytometry

In the analytical results section, we examined the relationship between various factors. While evaluating the correlation between the percentage of prolymphocytes and 6 cell markers including CD19, CD19&CD5 co-expression, CD20, CD22, CD23, and FMC7 no significant relationship was found (*P*>0.05). 

During the analysis, a significant relationship was observed between the percentage of atypical lymphocytes and the percentage of expression of cellular markers. An inverse relationship was identified between the expression of the FMC7 marker and the percentage of atypical lymphocytes (*P*=0.006). Furthermore, a weaker yet still significant direct relationship was found with CD19 (*P*=0.039), CD20 (*P*=0.021), and an inverse relationship with CD22 (*P*=0.032).

During the analysis of the relationship between CLL type and bone marrow involvement pattern, no significant correlation was discovered (*P*>0.05).

According to the results of the statistical analysis, a significant inverse relationship was found between the degree of bone marrow iron storage and the percentage of atypical lymphocytes (*P*<0.001). This indicates that as the percentage of atypical lymphocytes increases in peripheral blood among CLL patients, their bone marrow iron storage level decreases.

Based on the results of the statistical analysis, no significant correlation was observed between the type of CLL (typical/atypical), the WBC count, the percentage of prolymphocytes and lymphocytes in peripheral blood, thrombocytopenia and anemia with the degree of iron storage in bone marrow aspiration (*P*>0.05). 

Regarding the results of the statistical test, no significant relationship was found between anemia and the bone marrow involvement pattern (*P*>0.05). Similarly, the correlation between leukocytosis and thrombocytopenia with the pattern of bone marrow involvement was not statistically significant (*P*>0.05).

Finally, according to the outcomes of the statistical analysis, the mean percentage of CD22 and FMC7 marker expression on lymphocytes from individuals diagnosed with atypical CLL exhibited a higher value, although this difference lacked statistical significance. In contrast, the mean expression of CD23 on lymphocytes from those with typical CLL was notably elevated, showing a significant distinction (*P*=0.045). Regarding the remaining markers, no noteworthy correlation was detected.

## Discussion

Chronic lymphocytic leukemia (CLL) is a lymphoproliferative disease in which small and mature B lymphocytes are scattered in the blood, bone marrow, and sometimes lymph nodes. In cases involving lymph nodes with or without bone marrow engagement, the disease is called small lymphocytic lymphoma or SLL. In both of these diseases, neoplastic lymphocytes express the surface markers CD5 and CD23, and the expression of CD10, FMC7, and CD79b is decreased in them (24).

CLL/SLL is the most common type of leukemia in Western countries, affecting mostly elderly individuals and being rare in those under the age of 30. The ratio of men to women in this disease is 2:1, and the genetic sequence plays a role in its occurrence, with a probability that is 8 times higher in first-degree relatives (25).

In this study, 161 patients were enrolled with a definitive diagnosis of CLL, out of which 111 were male patients and 50 were female, resulting in a ratio of 2.2/1. This ratio is closely aligned with the findings of most studies. Specifically, Marzieh Bagheri's study reported a ratio of 2.1/1 (26), while Basabaeen's study reported a ratio of 2.5/1 (27).

The average age of the population was 63.8±10.8, with a median of 64 years. The age range at the time of diagnosis varied from a minimum of 30 to a maximum of 89 years. This is in comparison to the findings of Bagheri's study, which reported an average age of 64.34 (26), Payandeh's study with an average of 60.73 (28), and Basabaeen's study with an average of 62.97±12.06 (27).

Regarding the level of iron storage in the bone marrow, graded from 0 to 5, among the 131 patients for whom iron levels could be measured, the distribution of CLL patients according to the iron storage grades was as follows: grade 0 (7 patients), grade 1 (27 patients), grade 2 (37 patients), grade 3 (35 patients), grade 4 (19 patients), and grade 5 (6 patients). The discussion on evaluating bone marrow iron storage and its relationship with leukemias, including CLL, is a relatively new topic, and there have been limited similar studies conducted both within and outside of Iran. However, in a study conducted by Marjan Abedi on pediatric patients with acute lymphoblastic leukemia (PALL), the impact of iron content in bone marrow cells on their resistance to methotrexate treatment was examined. The study found that higher levels of iron in bone marrow cells correlated with increased resistance to methotrexate treatment. Consequently, elevated levels of iron in the body and bone marrow could potentially act as a factor against successful methotrexate treatment, potentially leading to disease relapse following treatment (29).

Among 158 patients with CLL at the time of diagnosis, 94 (59.4%) had anemia, while 64 did not have anemia. Bagheri's research findings indicated that 57.1% of patients in their study presented with anemia (17), whereas Basabaeen's investigation revealed a prevalence rate of 34.5% among the patients under examination (27).

Among the 160 patients diagnosed with CLL, 31 patients (19.3%) had thrombocytopenia at the time of diagnosis. The remaining 129 patients had normal platelet counts, with an average platelet count of 140,000. Bagheri's study reported a thrombocytopenia rate of 31.4% (26), while Basabaeen's study found that 39% of patients had thrombocytopenia (27). In Payandeh's study, the average platelet count among patients was 160,000 (28).

In terms of the pattern of bone marrow involvement, among the 159 patients, the frequencies of each pattern were as follows: interstitial pattern in 26 individuals (16.3%), focal pattern in 8 individuals (5%), diffuse pattern in 75 individuals (47.1%), nodular pattern in 21 individuals (13.2%), interstitial and nodular pattern in 12 individuals (7.5%), and interstitial and focal pattern in 17 individuals (10.6%). In Jahic's study of 40 patients, the distribution of bone marrow involvement patterns was as follows: 45% (18 patients) had a mixed pattern, 17.5% (7 patients) had an interstitial pattern, 10% (4 patients) had a nodular pattern, and 27.5% (11 patients) had a diffuse pattern (30). In Zengin et al.'s study involving 70 CLL patients, 48.6% (34 patients) exhibited a diffuse pattern, while the remaining 51.4% (36 patients) displayed a non-diffuse pattern, which consisted of 14 nodular, 11 interstitial, and 11 mixed cases (31).

In the present study, the average white blood cell count was 50,928±61,371 per microliter, with a median of 30,000, a maximum of 500,000, and a minimum of 6,300. Bagheri's study reported an average WBC count of 45,000 (26), while Basabaeen's study reported an average of 92.86 ± 75.43 x 10^3/μL (27).

The average percentage of lymphocytes in our study was 55.9 ± 28.2%, and the average absolute number of peripheral blood lymphocytes was 27,628± 39,716/μL. In Basabaeen's study, this figure was 82,230 ± 70,880/μL (27), and in Bagheri's study, the average number of patients' lymphocytes was 35,900/μL (26).

As shown, the results of our study concerning demographic parameters, such as age and gender, as well as the numbers of lymphocytes, anemia, and thrombocytopenia, align closely with findings from other studies, particularly those conducted domestically. In the following section, we will discuss the analytical results of the study.

In our study, we observed a significant correlation between the percentage of atypical lymphocytes in peripheral blood and certain markers. The increase in the percentage of atypical lymphocytes showed a weak association with the upregulation of CD19 and CD20 markers, with P-values of 0.039 and 0.021, respectively, and a decrease in the expression of FMC7 and CD22 markers, with p-values of 0.006 and 0.032, respectively. In a study conducted by Frater and colleagues, patients with a percentage of atypical lymphocytes above 10% were categorized as having atypical CLL. When comparing them to patients with less than 10% atypical lymphocytes using flow cytometry, it was found that patients with a higher percentage of atypical lymphocytes in peripheral blood had higher and brighter CD23 expression on their lymphocytes. However, no significant relationships were observed with the expression levels of CD5, CD19, and CD79b (32).

In our study, we found no significant relationship between the type of CLL and the pattern of bone marrow involvement. However, in Jahic's study, it was concluded that a significant relationship exists between the pattern of bone marrow involvement and the clinical stage of the disease. Specifically, patients in stage B or C of the Binet system at the time of diagnosis exhibited a higher prevalence of the diffuse pattern. This pattern was associated with disease progression and a worse prognosis. In contrast, the study by Zengin et al. did not reveal a strong significant relationship between the pattern of bone marrow involvement and the disease prognosis (30, 31).

In terms of the survey of the relationship between anemia and thrombocytopenia in CLL patients with the pattern of bone marrow involvement, no significant relationship was observed in our study. However, in Jahic and colleagues' study, as anemia and thrombocytopenia are criteria for stages B and C of the Binet system, an association was identified between these two conditions and the diffuse pattern of bone marrow involvement (30).

Finally, we examined the relationship between the types of CLL (typical or atypical) and specific cell markers. In our study, we did not observe any significant correlation between the type of CLL and the six-cell markers we investigated. Notably, the average expression of CD22 and FMC7 was higher in the atypical CLL group. Conversely, the simultaneous expression of CD5/CD19 showed a higher average in the typical CLL group. A weak but significant correlation emerged between the type of CLL and the CD23 marker. This finding indicates that the average expression of CD23 in patients with typical CLL was higher than in patients with atypical CLL (p-value = 0.045), aligning with the results of Frater and his colleagues' study, where CD23 expression was significantly higher in patients with typical chronic lymphocytic leukemia (*P*=0.04) (23). These findings shed light on the relationship between CLL types and specific cell markers, contributing to our understanding of the disease. As mentioned in Naeem's study, typical CLL is characterized by the expected proper expression of CD5, CD19, and CD23, moderate expression of CD20, and weak expression of CD22. Furthermore, CD79b and FMC7 are typically weak to negative in typical CLL cases (25). This is why we classified patients with positive expression of FMC7, CD79b, CD22, and/or weak expression of CD5, CD19, and CD23, who were diagnosed with CLL, into the atypical group.

## Conclusion

it is advisable to undertake prospective evaluations in patients with chronic lymphocytic leukemia to evaluate factors such as survival rates, treatment progression, and the clinical course of symptoms. Such studies can help further elucidate the relationship between laboratory findings and disease prognosis.
